# Ultrasonic atomizer based development of pH sensor for real time analysis

**DOI:** 10.1038/s41598-020-68005-2

**Published:** 2020-07-02

**Authors:** Gaurav Pandey, Sandeep Choudhary, Rashmi Chaudhari, Abhijeet Joshi

**Affiliations:** 1 Room No. SB-216, Discipline of Biosciences and Biomedical Engineering, Indian Institute of Technology Indore, Khandwa Road, Simrol, Indore, Madhya Pradesh 453552 India; 2 Discipline of Biosciences and Bioengineering, Indian Institute of Technology Bombay, Powai, Mumbai, India

**Keywords:** Biological techniques, Nanobiotechnology, Sensors and probes, Optical spectroscopy

## Abstract

Fluorescent pH biosensors have gained importance owing to their low cost utilization in real time monitoring of biological and food samples in comparison to conventional pH meters. The research reports a novel method of ultrasonic atomization for developing a fluorescent pH sensor for real-time analysis made of Fluorescein isothiocyanate (FITC)-dextran/FITC-dextran-Tris (2, 2′-bipyridyl) dichlororuthenium (II) hexahydrate as indicator and reference fluorophores, respectively. The process of ultrasonic atomization ensures formation of monodisperse dye immobilized alginate microspheres ensuring efficient pH sensing. The developed biosensor was tested on milk samples, which has a short life span and shows a significant fall in pH with time due to microbial spoilage. The proposed biosensor showed a linear range of pH 4–8 (R^2^ between 0.96–0.99 for different single/dual fluorophore biosensors) which suitably cover the pH of milk during the entire storage period and spoilage. The % recovery for predicted pH falls between 90–110% compared against standard pH meter, indicating a good accuracy of estimation and low turnaround time (10 min). Thus, real-time monitoring using fluorescent pH biosensor for milk samples may profoundly improve the economics of losses occurring in processing and storage with capability of in-package continuous quality assessment.

## Introduction

Biologically derived molecules are the most sought entities for the scientific community in developing the biosensors owing to their environment-friendly nature and biological specificity. From the first biosensor by Clark and Lyons, biosensors have been deployed for almost every field in research and industry which are directly or indirectly associated with healthcare and life sustainability^1^. Biosensors typically utilize different kinds of biological samples, food samples and environmental samples for detection of several health care anomalies, food contaminant detection or pollution detection. pH can be a very common deterministic variable to be used as a marker for any of these areas in a situation-specific manner to identify the pathophysiology in disease diagnosis, level of contaminants, the progress of reactions, stability of products, adulterations, pollutants etc.^2^. For example, milk is a ubiquitous nutritional food component which has high importance and is subjected to pH-induced degradation due to microbial growth and lactic acid production. Various types of milk proteins and dissolved solids make up a vital buffer system of milk which changes drastically as the microbial led spoilage increases. Thus pH determination in milk samples becomes a critical factor in determining the spoilage associated with degradation of milk-sample, which can very easily be monitored using different pH based detection systems^3^.

Newer advances in pH measurement modalities are being investigated for efficient management in several areas. An example of a modified ion-sensitive metal–oxide–semiconductor electrode was developed for pH sensing. It describes a pH sensor with Indium Nitride (InN) nanorod based electrode against Ag/AgCl as reference electrode to measure standard pH buffer solutions which showed that with reference electrode voltage of 3 V. The measured change in pH for a scale of 4–10 is reported in terms of change in drain-source current (IDS), with pH 4 having the top spot and pH 10 bearing the lowest value^4^. Another ionic potential and fluorescence-based fused pH sensor takes a combined approach of differential charge based shift, generating electrons of a different wavelength. The shift of ionic potential is the first deterministic factor of pH, then the ratio of excitation and emission light is another deterministic factor for pH values detection^5^. There are several recent developments in the field of optical approach for signalling and sensing for highly portable wearable biosensors for wide usage. Numerous healthcare and analytical testing problems are being targeted through mobile biosensing platforms known as lab on a chip or point-of-care testing (POCT) devices^2,6^.

Literature reports several fluorophores either alone or in combination like Semi-naphtha-rhodafluor (SNARF), SNARF-1-dextran, *N*-allyl-4-(4′-methyl-piperazinyl)-1,8-naphthalimide (AMPN) and meso-5,10,15,20-tetra-(4-allyloxyphenyl)porphyrin (TAPP), *N*-allyl-4-(4′-methyl-piperazinyl)-1,8-naphthalimide (AMPN), 5-(and 6)-carboxyfluorescein, 2′,7′-Bis-(2-carboxyethyl)-5-(and-6-)carboxyfluorescein (BCECF), 2′,7′-bis-(2-carboxypropyl)-5-(and-6-)-carboxyfluorescein (BCPCF), 5- (and 6)-carboxynaphthofluorescein, Anthofluorescein, Borondipyrromethene (BODIPY), Flubida-2, Flubi-2, Fe3O4 nanocrystals-MMP-9-*N*-carboxyhexyl derivative of 3-amino-1, 2,4-triazole-fused 1,8-naphthalimide (ANNA, 1,1-dimethyl-2-[2-(quinolin-4-yl)vinyl]-1H-benzo[e]indole (QVBI), NIR-Benzothiazole, Sulfonated pentamethine, septamethine cyanine, bacteriophage particles, carbon dots, quantum dots etc., have been utilized for pH biosensor development related applications^7–10^. Use of reference fluorophores in addition to a pH-sensitive fluorophore in the form of ratiometric biosensors is widespread to avoid sensor assay based variations^11^. A report mentions triple fluorophore combination as Fluorescein, Oregon green and Cy5 for pH sensing, where Cy5 acts as a reference dye. This tri-fluorophore setup has been deployed to assess lysosomal pH inside the cell to determine the rate of transfection^12^. FITC is a high-value pH-responsive fluorophore due to its p*K*a values in near-physiological ranges and amenability to attach to biomolecules through the iso-thiocyanate functional group^13^. FITC has also been immobilized in human red blood corpuscles (RBCs) ghost structures for pH sensing in detecting chloride-bicarbonate equilibrium of RBCs^14^. Macromolecular Fluorescein-dextran (FITC-dextran) has also been described for simultaneous determination of pH, urea, acetylcholine and heavy metals along with TRITC-dextran using biological recognition units like urease and acetylcholinesterase encapsulated in a sol–gel matrix^15^. The mode of encapsulation of macromolecular dyes is critical in maintaining the stability of the biosensors. Several polymers and carriers like silica aerogels, poly (diallyl dimethylammonium chloride) (PDADMAC)-poly(3,4-ethylene dioxythiophene) polystyrene sulfonate (PSS), poly-(*N*-3-aminopropyl pyrrole-co-pyrrole) (PAPCP), methacrylic-acrylic membranes, poly(*n*-butyl acrylate) (PnBA) membrane, poly(2-hydroxyethyl methacrylate) (pHEMA), mercapto propionic acid (MPA), mercaptoacetic acid (MAA), or thioglycolic acid (TGA), Poly-lactide-co-glycolide (PLGA), Poly-lactic acid (PLA) have been utilized for encapsulation of dyes and biological recognition units together to form a biosensor^9,16–19^. Natural polymers like alginates which are polymers of mannuronic-glucuronic acid units have already been proved to be excellent carriers for encapsulation of biologically active macromolecules considering their mild crosslinking conditions, porosity and ease of producing them in different forms like microparticles, nanoparticles, gels, films, sponges etc. Alginate is also commonly investigated for encapsulation of macromolecules like drugs, enzymes, proteins, peptides etc.^20–24^.

The current research aims to develop and optimize a composition of pH biosensor based on FITC-dextran encapsulated in alginate microspheres. Macromolecular FITC-dextran with different conjugation ratios and molecular weight were investigated for an optimized biosensing performance. Encapsulation of FITC-dextran 150 kDa and 500 kDa in alginate microspheres was achieved using a novel, facile encapsulation method of ultrasonic atomization and simultaneous ionic crosslinking. Single fluorophore based biosensor was then transformed into a dual fluorophore ratiometric biosensor using Tris (2,2′-bipyridyl) dichlororuthenium (II) hexahydrate (Rubpy) by ionic crosslinking. The biosensing assay microparticles were utilized for milk spoilage detection in an induced pH formulation and change in pH during storage. Literature reports several pH biosensors based on changes in specific ionic conductance or potential difference and current values of the solution. Till date, there are no reports for the application of ultrasound wave based atomization for pH biosensor development in an environment friendly manner and generation of monodisperse fluorescent pH biosensor based on FITC-dextran-alginate microspheres. The proposed biosensor also demonstrates greater feasibility in the context of synthesis, cost effectiveness, environment friendly features scale up, deployment and real time analysis for quality assessment in different food samples esp. milk. This reflects in terms of monodispersity of microspheres leading to increased accuracy, low turn around time and longer shelf life in comparison to previously reported biosensors like those developed by air driven atomization^17,25^.

## Materials and methodology

### Materials

Sodium-alginate, fluorescein isothiocyanate–dextran (FITC Dextran 150 kDa and 500 kDa), Tris (2,2′-bipyridyl) dichlororuthenium (II) hexahydrate (Rubpy) were procured from Sigma Aldrich, India. Calcium chloride, trisodium citrate dihydrate, sodium carbonate and sodium bicarbonate were procured from Rankem, India. Citric acid monohydrate and citric acid anhydrous from S D Fine-Chem Ltd, India, while Tris hydroxymethyl methylamine from Fisher Scientific, India. All the chemicals used were analytical grade and used without any processing or purification. Milk samples were procured from the local rural market at the vicinity of IIT Indore, India and was used without any treatment.

### Ultrasonic atomizer based development and optimization of pH sensors

Alginate microspheres containing FITC-dextran [150 kDa (FITC:glucose, 1:160) and 500 kDa (FITC:glucose, 1:100)] were formed using a method of ultrasonic atomization (Sonaer, USA). A spraying mixture containing FITC-dextran 150 kDa/FITC-dextran 500 kDa (1 mg/ml) in Sodium alginate (0.7% w/v) was prepared in deionized water and pumped through a syringe pump (New Era Pump Systems NE-1000, USA). The spray of this mixture was collected in a crosslinking solution of CaCl_2_ (4% w/v) in deionized water with continuous mixing. Different parameters like concentration range, flow rate (18 ml/h), atomizing frequency (130 kHz) with over 90% power, stirring speed, and distance of crosslinking solution were optimized to obtain alginate microspheres of desired shape and size. The loaded alginate microspheres-FITC-Dextran 150/500 kDa (AM-FD150, AM-FD500) so formed were centrifuged and washed at 7000*g* in triplicate to remove excess CaCl_2_ solution and unloaded dye macromolecules. To develop the dual fluorophore, ratiometric pH biosensor, Tris (2,2′-bipyridyl) dichlororuthenium (II) hexahydrate (Rubpy) solution (1 mg/ml) was used as a reference fluorophore. It was incubated with AM-FD150 and AM-FD500 for about 30 min, and then the unbound dye was washed in triplicate using centrifugation at 7000*g* with deionized water. The purified microspheres were suspended in a small volume of deionized water and stored in refrigerated conditions until used. Stability of biosensor for its shelf life was evaluated with standard buffers used in biosensing protocol, in terms of activity per cent from the day of synthesis termed as zero-day. The activity of biosensor was assessed on consecutive intervals (days) with the same set of buffers in comparison to the activity level from day zero.

### Characterization

Alginate microsphere-based biosensors were characterized using different techniques like optical microscopy, scanning electron microscopy (SEM) and confocal microscopy (CLSM). Morphology, particle size and distribution of biosensor microspheres population was imaged by dropping a few drops on a glass slide using optical microscopy at a magnification of 10 ×–80 × using (Leica S8APOEC3, Germany) with an attached camera. The particle size distribution in an optical microscopy image was confirmed from ImageJ software which is open-source Java-based image processing software developed at the National Institutes of Health (NIH) and the Laboratory for Optical and Computational Instrumentation (LOCI, University of Wisconsin). After scale was calibrated to pixel length, the colour image was converted to grayscale, and after appropriate thresholding, particle size distribution analysis was represented as a histogram. Alternatively, the microspheres were also studied and imaged using a Field Emission Scanning Electron Microscope (FE-SEM Supra55 Gemini, Zeiss Germany) for their morphology and structural parameters. For FE-SEM imaging a 20 µl biosensor microspheres are dried on a slide, over silica gel and coated with gold or copper for increasing the conductivity by sputter coating (Quorum technologies Q150R, England) and visualized at different magnifications. Distribution of different fluorophores inside the alginate microspheres was visualized using Confocal Laser Scanning Microscopy (CLSM), (Olympus, Japan) with oil immersion at 100 × magnification. The excitation wavelength for both single and ratiometric fluorophores was at 488 nm so that FITC-dextran and Rubpy can be excited simultaneously. Green and red filters in the CLSM were used for imaging of FITC-dextran (λ_em_ 520 nm) and Rubpy (λ_em_ 610 nm), respectively. A calibration curve was developed in the concentration range of 0.01–0.1 mg/ml for calculating encapsulation efficiency of FITC-dextran 150 kDa and 500 kDA by indirect method for analyzing supernatants using UV spectroscopy (Perkin Elmer LAMBDA 325, USA) at 488 nm. FTIR analysis of samples like FITC-dextran, alginate and AM-FD 150 were performed using an FTIR spectrometer (Bruker, vertex 70 Germany).

### Biosensing studies

Biosensing studies were performed by exposing different standard pH buffers (in the range of pH 3–10 prepared using a standard pH meter (Hanna Instruments HI 8424, Romania) to alginate microspheres and capturing fluorescence emission scans between 505–650 nm at an excitation wavelength of 488 nm. Standard pH buffers used in testing the biosensors microspheres were developed using citric acid-sodium citrate (0.1 M) (pH 3–6), HCl-Tris Base (0.2 M) (pH 7–9) and Sodium carbonate-sodium bicarbonate (0.2 M) (pH 10). The biosensing assay constituted of 1 ml of buffer solutions mixed with 500 µl of biosensor microparticles. After a short incubation of 10 min approximately, fluorescence emission scans were captured using a fluorescence spectrometer (Horiba Fluorolog, USA). Stability of biosensor for its shelf life was evaluated with standard buffers used in biosensing protocol.

### Milk spoilage detection using developed pH biosensors

Milk samples were tested with the developed biosensors to detect their efficacy in determining the pH changes in both buffers induced samples and time-based storage and spoilage. Milk samples (1%, 10% and 20%) were mixed with biosensors micro-particles in different buffers (1:2) were combined to create induced pH changes in milk samples and analyzed using fluorescence spectrophotometer at an excitation wavelength of 488 nm. Similarly, the time-dependent samples in comparison were collected every 3 h for 24 h to be assessed with the biosensor and conventional pH meter simultaneously. Time-based degradation of milk was studied by analyzing the pH changes happening in milk using the single/ratiometric pH biosensors without any buffer added like the previous step.

### Statistical analysis

The statistical analysis of the experiments was performed using the Pearson correlation coefficient (r) to find the correlation between the predicted and actual pH values and two-way ANOVA to find the statistical significance between the different pH ranges and biosensors by IBM Statistical Package for Social Science (SPSS), Version 25, software. Predictions of milk pH by different biosensors using a fluorescence spectrophotometer against the actual values from the laboratory pH meter were compared using these tests to understand the statistical significance^26^.

## Results and discussion

### Ultrasonic atomizer based development and optimization of pH sensors

**Figure 1:**
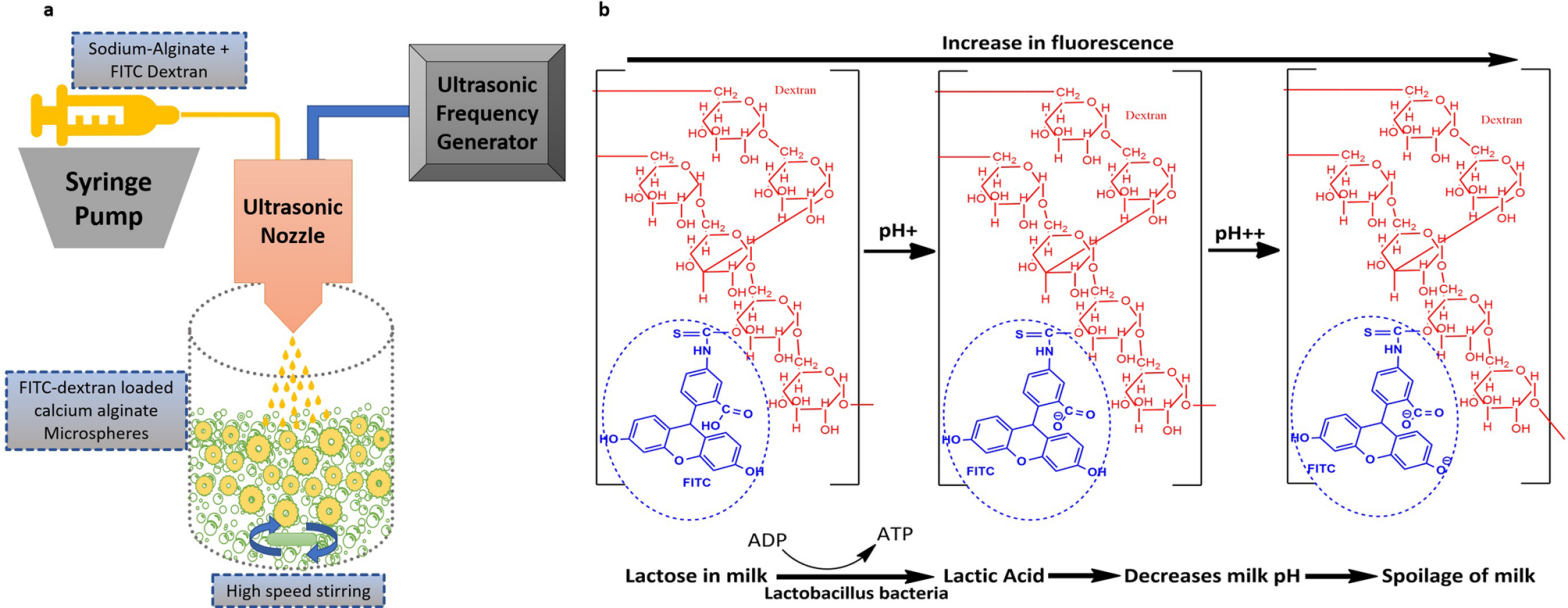
Schematic of the process of development of pH biosensors using an ultrasonic atomizing system (**a**). Mechanism of structural changes and ionization in different pH condition leading to generate different fluorescent states, for FITC-dextran in relation to changes in milk pH causing pH detection (**b**).

FITC-dextran used in the prepared pH biosensor acts as a macromolecular pH-sensitive fluorophore which ionizes in higher pH range with its conversion of mono and di-anionic forms at carboxylic and hydroxyl groups. The di-anionic form of FITC leads to an exponential rise in fluorescence emission intensity with alkaline pH (Fig. 1b)^27^. FITC in the macromolecular form was chosen to have a stable encapsulation in polymeric carriers like alginate as stability of biosensor performance is a critical function of molecular weight of dyes and consequent leaching of dyes. Macromolecular FITC, i.e., FITC dextran 150 kDa and 500 kDa can be more suitable for improving biosensor performance owing to reduced leaching from encapsulated system^27^. The entrapment of FITC dextran dye is highly dependent on the available inner space of the formed spherical structure of Ca-alginate. Alginate polymer utilized here is a known polymer to encapsulate several biologically active molecules like drugs, dyes, proteins, enzymes etc.^28–30^. It is a suitable polymer considering its mild ionic induced crosslinking conditions, and due to porous nature, small ions and analytes can permeate outside to inside, and byproducts can permeate outside from inside. Rubpy is another fluorophore added in the single fluorophore microspheres so that it acts as reference fluorophore as it has been known for its pH insensitive properties. Rubpy being a positively charged entity interacts with negatively charged alginate microspheres. Ultrasonic atomizer works on the phenomenon of ultrasonic vibrational pulses created by the ultrasonic frequency generator. Small droplets generated through ultrasonication depends on several parameters like concentrations of polymer and cross-linker, flow rate, frequency, power input, the ratio of spraying volume and collection volume, the distance between the spraying nozzle and collection surface, stirring rate etc. Atomized spray generated from an ultrasonic nozzle (Sonaer, USA) is crosslinked with a well-mixed CaCl_2_ solution forming calcium alginate microspheres (Fig. 1a). Particle size and morphology of alginate microspheres were investigated and optimized, which are summarized in Table S1. Table S1 suggests that flow rate, the concentration of alginate solution and volume ratio produces interplay of properties to provide a precise particle size distribution. Principally, a reduction in particle sizes is a result of increasing the frequency and power of ultrasonic atomization, decreasing the flow rate and volume ratio of polymer to cross-linker. A spraying solution (0.7% w/v alginate, 1 mg/ml FITC-dextran) and collecting solution (4% w/v CaCl_2_) with the volume ratio of 2 ml:50 ml, for contact distance of 5 cm and stirring rate of 1,000 rpm approximately were chosen as optimized protocol considering small particle size and close range of size distribution. Accuracy of the reported biosensor is very close to standard pH meter, but due to its optical approach for sensing it does not involve much physical contact like a conventional pH electrode. Which shows a higher significance of this experimental work in the area of analytical testing and diagnostics.

### Characterization

**Figure 2:**
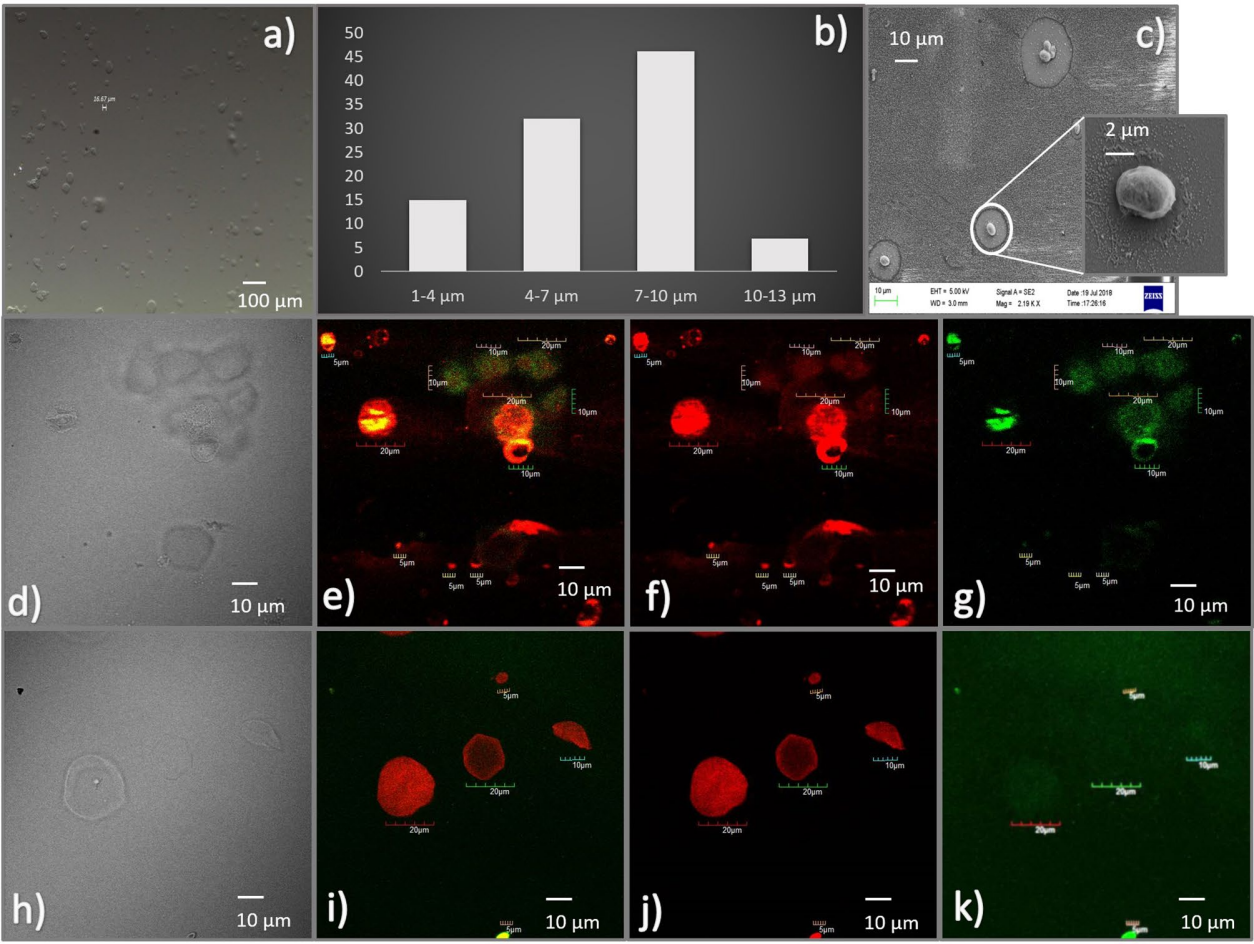
Characterization of FITC-dextran/FITC-dextran-Rubpy loaded alginate microspheres: optical microscopic images (**a**), Histogram describing particle size distribution (**b**), FE-SEM images (**c**), and CLSM images of AM-R-FD150 (merged) (**e**), AM-R-FD150 (red filter) (**f**), AM-R-FD150 (green filter) (**g**), AM-R-FD500 (merged) (**h**), AM-R-FD500 (red filter) (**j**), AM-R-FD500 (green filter) (**k**) and corresponding DIC images (**d**, **h**).

Particle size and distribution were studied using optical microscopy (Leica, Germany). Optical microscopic imaging shows that it was possible to measure particle sizes within the size range of 5–20 µm (Fig. 2a). The average particle size distribution of particle population with ImageJ, open-source software for image processing showed a particle size distribution in the range of 1–15 µm based on the differential interference contrast (DIC) microscopy image. A histogram describing the particle size distribution shows the diversity of particles produced. The results show that from the total population size, approximately 15% population belongs to 1–4, 32% belong to 4–7, 46% belong to 7–10, and 7% belong to 10–13. This observation explains that a majority of 78% of particles belongs to the size group of 4–10 (Fig. 2b).

**Figure 3:**
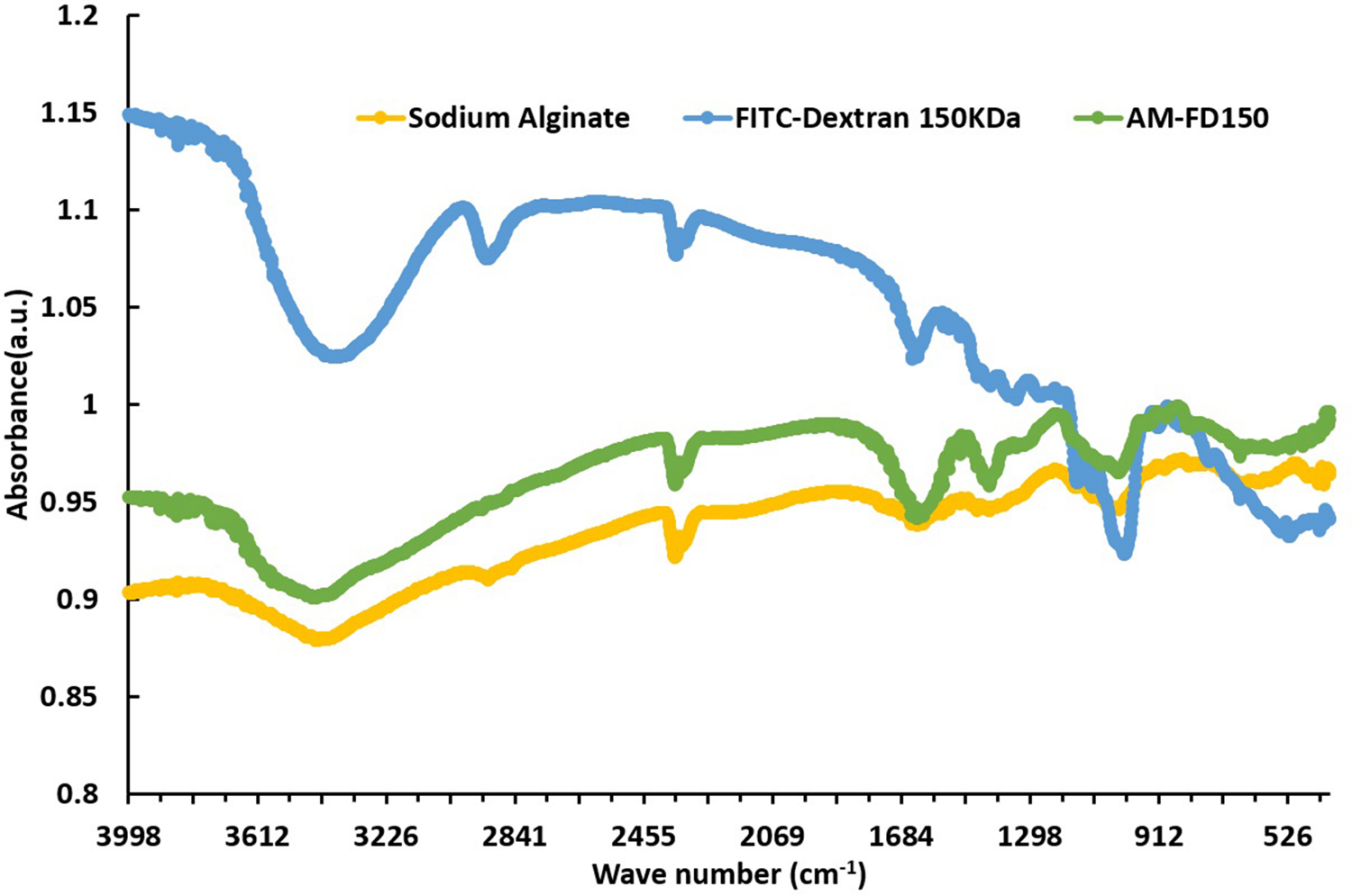
Fourier-transform infrared spectroscopy (FTIR) analysis of individual alginate, and FITC-dextran 150 kDa in comparison to pH biosensor.

FE-SEM studies were also focused towards determining particle size, distribution and surface morphology of biosensor particles. The images indicate that spherical and porous in morphology forming small clustered groups of particles then were observed with sizes in the range of 3–10 (Fig. 2c). Further analysis on a higher magnification, particle size distribution was found to be approximately 3–10, where most of the particles are have sizes of 3–7, (inset of Fig. 2c). The biosensors formulations (AM-FD150, AM-R-FD150, AM-FD500 and AM-R-FD500) were imaged using CLSM imaging at 100 × with oil immersion lens setup using green and red filters. The different filtered images for both dyes were superimposed to illustrate the overall distribution inside the particles as co-distribution of FITC and Rubpy (Fig. 2d–g and h–k). CLSM images showed that higher localization of FD and Rubpy inside the noticed spherical boundaries of alginate microspheres. FITC-Dextran 500 kDa photo-bleached more readily in comparison to FITC-Dextran 150 kDa, which was also an effect produced due to dye loading capability (Fig. 2g, k). FITC-Dextran 150 kDa molecule is much smaller in comparison and being in the same concentration of spraying solution it will contain inhouse more units of dye than the FITC-Dextran 500 kDa version. The merged signal image of Rubpy with FITC-Dextran (Fig. 2e) showed that both the fluorophores are uniformly distributed inside the alginate microspheres. Our microspheres show a more precise particle size and distribution in comparison to previous methods of synthesizing alginate microspheres. When the encapsulation efficiency was evaluated for FITC-dextran 150 kDa and 500 kDa in alginate microspheres, results indicated encapsulation efficiency was found to be about 90 ± 5% and 80 (± 10%) with respect to initial theoretical loading, respectively. Figure 3 shows the characteristic differences between alginate polymer, FITC-Dextran 150 kDa. Alginate Microspheres housing FITC-Dextran 150 (AM-FD150) shares its scan characteristics with both of its constituent molecules. Free Alginate polymer shows the O–H bond, C–H bond stretching peaks at 3,700 and 2,900 range, while at 2,350 and 1,600 heights arises from C–H bond bending and C–O aliphatic ether bond stretching peak at 1,009 range. Free FITC-Dextran molecule shows the N–H bond stretching, S–H (thiol) bond peaks at 3,300 and 2,900 range, while at 2,300 and 1,600 peaks arises from C–H aldehyde bond, N–H bond bending and C–N amine bond stretching at 1,000 range. Interestingly, AM-FD 150 molecule shows the O–H bond stretching, C–H bending aldehyde bond at 3,500 and 2,300 range similar to alginate, C–H aromatic bond bending, C–N amine bond stretching peaks at 1,300 and 1,000 range similar to FITC-Dextran 150 molecule, while at 2,300 and 1,600 range the peaks are similar to both alginate and FITC-Dextran 150 molecules.

### Biosensing studies

**Figure 4:**
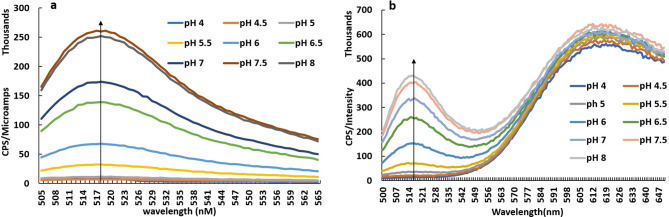
Typical overlay of fluorescence biosensing profile using standard pH buffers employing (**a**) single fluorophore pH biosensor, (**b**) dual fluorophore pH biosensor.

**Figure 5:**
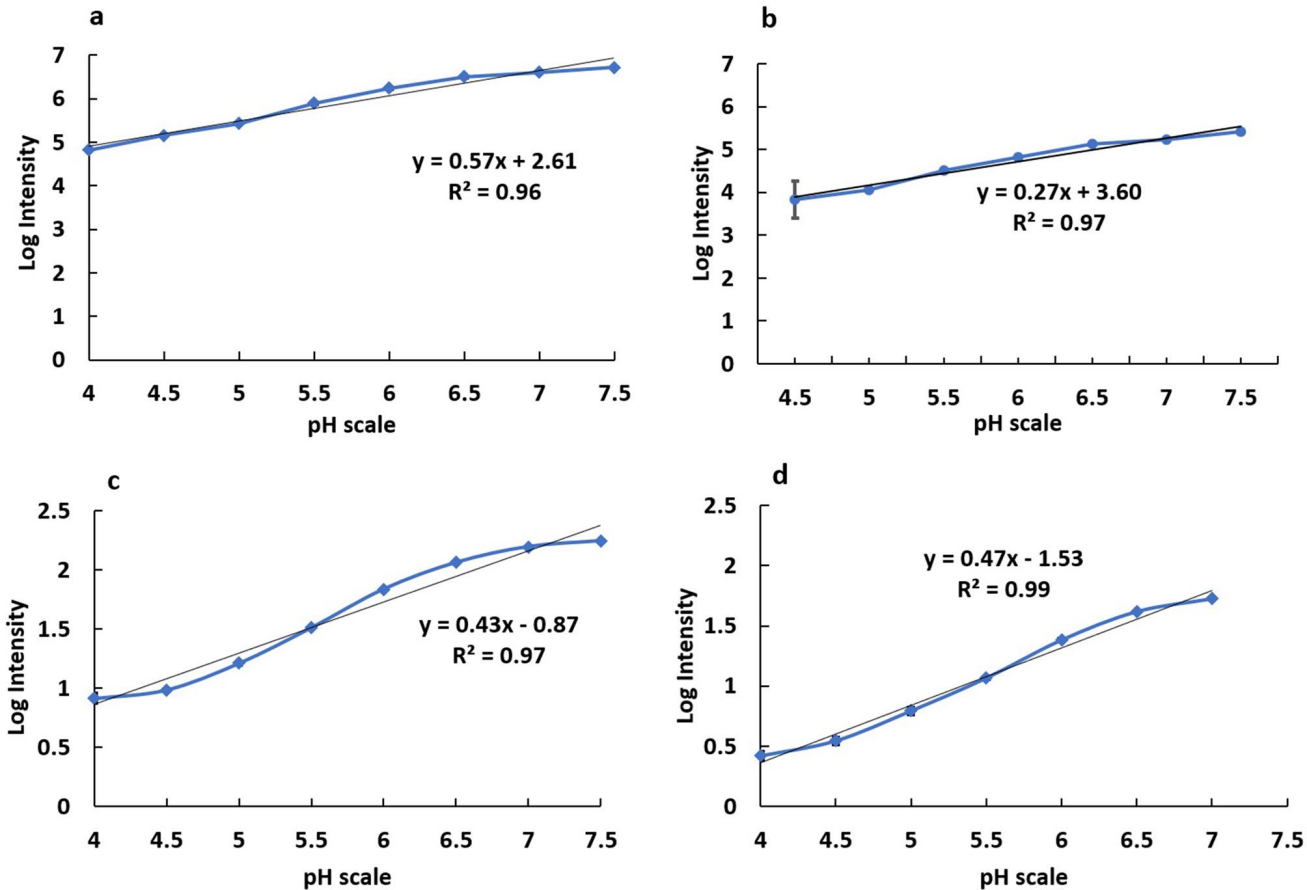
Linear regression analysis of logarithmic plots of fluorescence emission in response to changes in pH indicating linearity of pH biosensors with standard buffer solutions (**a**) AM-FD150, (**b**) AM-FD 500 kDa, (**c**) AM-R-FD 150 kDa, (**d**) AM-R-FD 500 kDa (error bars represent the standard deviation for n = 3 samples).

**Figure 6:**
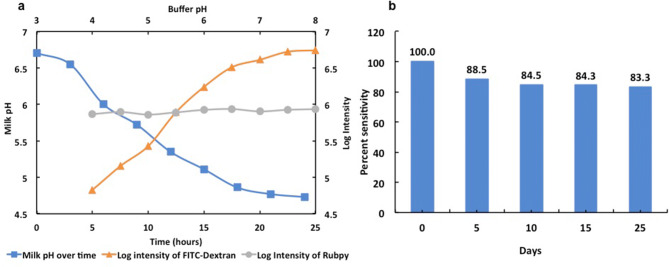
(**a**) pH profile of raw milk showing pH drop over time (primary axis), FITC-dextran fluorescence emission profile with different pH buffers (secondary axis) and Rubpy fluorescence emission profile with different pH buffers (secondary axis); (**b**) life-span of pH biosensor from the day of synthesis (i.e. 0th day representing 100% activity) in terms of percentage activity.

Biosensing studies of pH biosensor microspheres were deployed with standard pH buffers in the range pH 3–10 using a fluorescence spectrophotometer (Horiba Fluorolog, USA). Typical fluorescence emission spectra for AM-FD150 are described with an excitation wavelength 488 nm, show that emission intensity at 520 nm increases as the alkalinity increases between pH 4–8 (Fig. 4a, b). The AM-FD150 biosensor shows highly resolved sensing response from the range of pH 5 to pH 8, while the activity below pH 5 is less resolved due to poor ionization of FITC Dextran molecule when it exists as a cationic molecule form^13,31^. On the other hand, an overlay of AM-R-FD150 emission spectra for different pH also shows that increase in fluorescence intensity at 520 nm increases with increase in alkalinity and the emission at 610 nm of Rubpy remains almost constant which clearly shows its pH insensitive nature (Fig. 6a). The pH-sensitive units of FITC-dextran inside alginate microsphere emit fluorescent signal while being ionized inside pH solutions. The differences in ionic states and strength of ionization within standard buffer solutions of pH solutions between 4.5 to 7.5 pH value results in a gradient of intensities. This ratio of intensities are converted to the logarithmic plot, and a standard equation is derived for calibration of biosensing activity. The fluorescence emissions from a single fluorophore sensor’s activity are recorded from 500 to 565 nm, while for dual fluorophore the emissions from 500 to 650 nm has been documented through fluorescence spectrometer. The signal intensity values from a single fluorophore sensor are directly converted to the logarithmic plot. In contrast, for a dual fluorophore sensor, a ratio of Rubpy and FITC-Dextran emission have been taken and then converted to the logarithmic plot. A linear regression analysis of logarithmic plot of intensities for AM-FD150 and AM-R-FD150 showed a good regression coefficient R^2^ = 0.96 (y = 0.57x + 2.61) and R^2^ = 0.97 and (y = 0.43x − 0.87), respectively (Fig. 5a, c). Similarly, linear regression analysis of logarithmic plot of intensities for AM-FD500 and AM-R-FD500 also showed a good regression analysis R^2^ = 0.97 (y = 0.27x + 3.60) and R^2^ = 0.99 (y = 0.47x – 1.53), respectively (Fig. 5b, d). The dual fluorophore ratiometric biosensor with 500 kDa FITC-dextran performs less efficiently with a range of pH 4.5–7.5 in comparison to 150 kDa FITC-dextran (pH 4–7.5). In case of 500 kDa based biosensor, it seems that the AM-R-500 kDa works superior to AM-500 kDa when we compare the slopes of the standard curves. These variations in responses can be explained by the low encapsulated concentration of FITC-dextran 500 kDa due to space constraint in small particle size of 4–10 µm. This fact is in line with research published by Chaudhari et al. which describes high encapsulation of FITC-dextran 500 kDa in alginate microspheres (30–50 µm) formed using an air-driven atomization technique^30^. Rubpy encapsulation is mostly dependent on ionic charges and does not depend on space availability constraints. Table S2 describes a comparison of the sensitivity values of all the formulations. About, a 5–20% increase in sensitivity was observed when different pH ranges (pH 5–7.5 vs pH 4–8) are evaluated. Improved performance in pH ranges 5–7.5 may help our goal of monitoring the pH of milk samples, as changes in pH of milk also occur in the range pH 5–7.5. Below pH 5 milk has a higher propensity to degrade. In another aspect of applicability for our biosensor, is its stability. In storage condition and activity during the storage period has been a critical factor in the efficiency of pH biosensor. The shelf life was measured in the percentage of activity retained from the commencement day. In this study, the pH biosensor shows a good shelf life of approximately a month (30 days) with a retained activity of more 80% (Fig. 6b) from the day zero being 100% in its activity.

In both the FD-150 kDa and FD-500 kDa sensor formulations, the transformation to ratiometric sensors leads to a decrease in sensitivity of about 11%. This loss in sensitivity may be a cause of concern, but the process brings an added advantage of increased accuracy and preventing variability in sensing assay performance and stability. However, according to statistical two-way ANOVA test, there is no significant difference (p-value = 0.07) (p > 0.05) between the average sensitivity values (Table S2) for pH 4–8 and pH 5–7.5 in all the sensor formulations. Among all the sensors also, the sensitivity comparison shows no significant differences (p value = 0.17) (p > 0.05) in both pH 4–8 and pH 5–7.5. This indicates that any of the sensor formulations can work as a pH-based biosensor.

### Milk spoilage detection using developed pH biosensors

Milk is highly nutritious food product containing components like water (87%), proteins (3%), fats (3–4%), lactose (4–5%) necessary minerals (0.8%) and vitamins (0.1%) which has gained importance in growth and development of humans^32^. Due to the composition with essential nutrients, it also acts as a rich medium for bacterial growth and is highly prone to contamination and degradation. Degradation of milk is associated with a pH decrease due to microbial growth and lactic acid formation. The mapping and monitoring of such deterioration can be studied using the developed pH biosensors (AM-FD 150 kDa, AM-FD 500 kDa, AM-R-150 kDa and AM-R-500 kDa). Assessment of milk's quality and shelf life has been an essential question for industrial as well as regular dairy organizations. There are several experimental studies to address this problem in more than one way. Presence of some external components like antibiotics can also cause hindrance in the assay for shelf life. For example, since milk is rich in lactose sugar, hence a lactate dehydrogenase enzyme-based amperometric lactate biosensor has been reported by Rahman et al. to solve the problem; through sensing the lactate concentration electrochemically through NAD/NADH. Similarly, from another experimental study, pH sensing is aimed through a fused approach of a potential-based fluorescent sensor that combines the shift in the ratio of reference and input diode's electrode potential value and the proportion of excitation and an emission wavelength of generated electrons by Nakazawa et al. The overall idea appears to detect pH either by detection of a shift in potential or by the concentration of a causative analyte in the solution^5,33^.

Variations in pH for over 24 h was monitored to study a time-based degradation profile of milk sample, (Fig. 6a). The result indicates that pH of fresh milk was found to be in the range pH 6.5–6.8 and during storage after 24 h the pH value could drop to pH 4–4.5 due to formation of lactic acid and microbial growth leading to precipitation and coagulation of milk proteins. Figure 6a also shows that FITC has a similar drop of fluorescence emission with pH change that affirms suitability of FITC for predicting pH of milk samples. Similarly, Rubpy showed no changes in emission with pH change. Figure 3 describes the FTIR analysis of pH sensor formulations describing the integration of dyes in polymeric alginate carriers. Influence of pH on milk was studied by inducing pH changes pH 4 to pH 8 using standard buffers and testing the biosensor performance to calculate the accuracy of predicted pH, using a standard pH meter and the developed biosensor was evaluated using calibration curve equations like (y = 0.57x + 2.61), (y = 0.27x + 3.60), (y = 0.43x − 0.87) and (y = 0.47x − 1.53), respectively (Fig. 5a–d).

**Figure 7:**
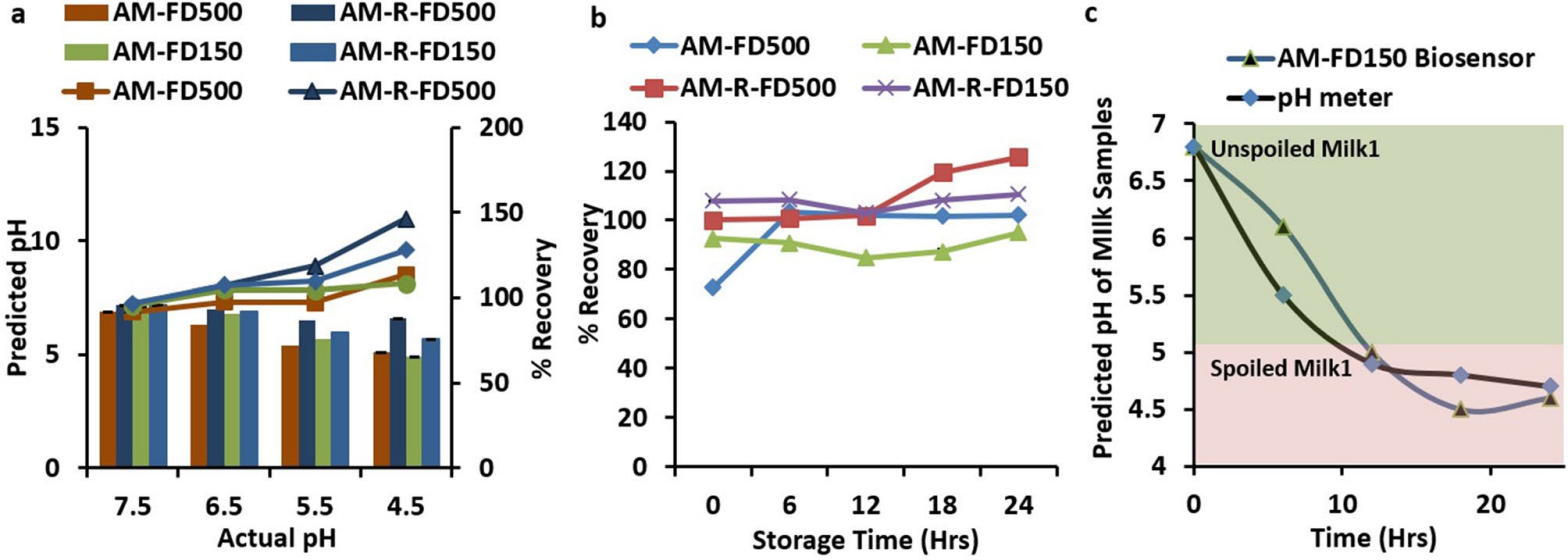
Accuracy studies of pH prediction using different pH biosensors when compared against a laboratory pH meter (**a**) [columns representing the predicted pH vs actual pH (primary axis) and lines representing % recovery vs actual pH in secondary axis], (**b**) % recovery values of pH prediction using different pH biosensors during storage and spoilage of raw milk (100% shows the accuracy close to pH meter); (**c**) spoilage prediction using AM-FD150 pH biosensor and pH meter showing the transition of milk spoilage from both analytical method.

The accuracy studies show that the % recovery values and Pearson correlation (r) values for different sensor formulations were found to be AM-FD 150 kDa (95–108%, 0.982), AM-FD 500 kDa (92–113%, 0.944), AM-R-150 kDa (97–127%, 0.976) and AM-R-500 kDa (96–146%, 0.817) (Fig. 7, Table S4). AM-FD 150 kDa showed no significant (0.982) difference between the actual and predicted pH values in comparison to other sensor formulations because of higher encapsulation efficiency of FD-150 kDa in comparison to FD-500 kDa. FD-150 kDa is a smaller molecule in comparison to FD-500 kDa and thus can easily be encapsulated in alginate microspheres. Between pH 5–7.5, all the biosensors demonstrated no significant difference and a comparable accuracy in the detection of pH. Although when considering ratiometric biosensors, AM-R-150 kDa provide better significance (r-value 0.976) in comparison to AM-R-500 kDa (r-value 0.817), however, it is observed that at pH 4.5 the ratiometric biosensors presented poor recovery values because of the low response of FITC below pH 5 which may have been colluded by poor loading of FITC-dextran 500 kDa.

When the accuracy of biosensors was tested over 24 h duration, FD 150 kDa was found to be more accurate and significant with % recovery values and Pearson correlation (r) of 84–95% and 0.986 for a single form and 103–110% and 0.991 for dual ratiometric form, respectively where as FD 500 kDa shows the % recovery and significance of 72–103% and 0.518 for single form and 100–126% and 0.939 for dual ratiometric form, respectively (Fig. 7b, c; Table S5). Among all the biosensors, a ratiometric form of FD-150 showed a higher significance 0.991 between the actual and predicted pH values and optimum % recovery (103–110%) (Fig. 7a). Higher variability or inaccuracy of detection was found to be a result of poor loading of dyes and lower level of effectiveness of FITC dye to pH values lower than 5. Figure 7b shows that the AM-FD150 biosensor can simulate and map the actual pH drop occurring during the spoilage of milk samples. A threshold pH of 5 can be assumed for complete decay to take place and thus directly predicting the fate of any milk sample whether it is prone for spoilage or not depending on the storage conditions. The results indicate that exponential drop in pH over 24 h duration can be suitably measured using a fluorophore based on FITC, which also has a similar exponential decrease in fluorescence emission with a fall in alkalinity. Thus, FITC-dextran based single or dual fluorophore-based biosensors can act as suitable platforms for pH detection of milk samples and simultaneous stability monitoring. Through these experimental results, we can state that our developed biosensor does not rely on the concentration of a specific analyte for assessing the pH like lactate biosensor, neither its activity gets affected by any extra component like antibiotics or others (Figure S1). Apart from the biochemical hindrance, our biosensor does not has the non-biodegradable materials that are not easily disposable. Here through our biosensor, we present an upgrade for the assessment of milk shelf life and edibility through a safely disposable environment-friendly approach, that possess almost similar accuracy as a standard lab equipment with real time sensing capabilities.

## Conclusion

pH determination has remained a valuable tool to determine the status and shelf life of several food products like milk. pH changes occurring in milk during storage can be determined using fluorescence-based pH biosensors. Thus, the current research deals with developing FITC-dextran and Rubpy encapsulated alginate microspheres which can serve as single and dual fluorophore pH biosensors. The method of development of these alginate microsphere-based biosensors was performed using ultrasonic atomization and ionic crosslinking. The alginate microspheres were formed with sizes in the range of 4–10 µm. The results have indicated that the tested biosensor formulations (single and dual fluorophores biosensors) show that the pH biosensor can work in a range of pH 4–8 with a linear response for logarithmic plots having R^2^ values higher than 0.95, which can support most of the physiological conditions. The biosensor shows that they can provide an accurate estimation of pH within a recovery range of 90–110% when compared using a standard laboratory pH meter. Among all the biosensor formulations tested FITC-dextran 150 kDa shows high sensitivity in comparison to the FITC-dextran 500 kDa counterpart. The dual fluorophore ratiometric biosensor, on the other hand, provided a slightly lower sensitivity. The results clearly indicate that pH biosensors can be utilized for accurate determination of shelf life of milk in a precise manner. The application of ratiometric nature of biosensors using two fluorophores will aid in to strengthen the credibility for deployment and usage desirably. Along with accuracy a good shelf life with an activity of more than 80% for approximately a month is an outstanding development for its efficient profile. Use of environment-friendly polymers also would be suitable for the safe disposal of these biosensors post usage. Thus, such fluorescent pH biosensors can act as promising tools to reduce the economic losses in post-harvest processing of milk.

## Supplementary information

Supplementary file1 (DOCX 393 kb)

## Abbreviations

DDI: Double deionized water
FITC Dextran: Fluorescein isothiocyanate–dextran
Rubpy: Tris (2, 2′-bipyridyl) dichlororuthenium (II) hexahydrate
AM-FD150: Alginate microspheres loaded with FITC dextran 150 kDa
AM-R-FD150: Alginate microspheres loaded with FITC dextran 150 kDa and conjugated with Rubpy
AM-FD500: Alginate microspheres loaded with FITC dextran 500 kDa
AM-R-FD500: Alginate microspheres loaded with FITC dextran 500 kDa and conjugated with Rubpy
