# Supplementary material for: Ultrasonic atomizer based development of pH sensor for real time analysis
Source: Sci Rep. 2020 Jul 2;10:10910. doi: 10.1038/s41598-020-68005-2 (PMC7331635; doi:10.1038/s41598-020-68005-2)
Supplement: Supplementary file 1 — Supplementary file1 (DOCX 393 kb) [file 41598_2020_68005_MOESM1_ESM.docx]

**Supplementary Information**

**Ultrasonic Atomizer based Development of pH Sensor for Real Time Analysis**

Gaurav Pandey^1^, Sandeep Choudhary^1^, Rashmi Chaudhari^2^, Abhijeet Joshi ^1,^*

^1^Discipline of Biosciences and Biomedical Engineering, Indian Institute of Technology Indore, Khandwa Road, Indore, India

^2^Discipline of Biosciences and Bioengineering, Indian Institute of Technology Bombay, Powai, Mumbai, India

**Table S1** Summary of optimization of development of calcium alginate microspheres using an ultrasonic atomizer

| **C**_alginate_  (% w/v) | Ratio (ml) **V**_alginate_ **:V**CaCl_2_ | Flow rate (ml/h) | Distance approx (cm) | Size of micro particles (µm) |
| --- | --- | --- | --- | --- |
| 0.3 | 2ml:30ml | 30 ml/h | 10 | 30 ± 10 |
| 0.3 | 2ml:50ml | 18 ml/h | 5 | 5 ± 10 |
| 0.5 | 2ml:30ml | 30 ml/h | 10 | 25 ± 10 |
| 0.5 | 2ml:50ml | 18 ml/h | 5 | 5 ± 10 |
| 0.7 | 2ml:30ml | 30 ml/h | 10 | 25 ± 10 |
| 0.7 | 2ml:50ml | 18 ml/h | 5 | 10 ± 05 |

CaCl_2_ concentration (4% w/v), Ultrasonic frequency: 130 KHz, Power settings 98%

**Table S2** Comparison of Sensitivity across the single and dual fluorophore biosensor profiles

| **pH** | **Sensitivity (%)** | | | |
| --- | --- | --- | --- | --- |
|  | **AM-FD150** | **AM-FD500** | **AM-R-FD150** | **AM-R-FD500** |
| 4-8 | 47.5 | 37.5 | 35.0 | 35.0 |
| 5-7.5 | 50.0 | 53.3 | 40.0 | 43.3 |

**Table S3** Comparison of Pearson correlation across the single and dual fluorophore biosensor profiles

| **Induced pH** | **AM-FD-150** | **AM-R-FD-150** | **AM-FD-500** | **AM-R-FD-500** |
| --- | --- | --- | --- | --- |
| Pearson Correlation | 0.982** | 0.976** | 0.944** | 0.817* |
| Sig. (2-tailed) | 0.000 | 0.000 | 0.189 | 0.013 |
|  |  |  |  |  |
| **Time dependent** |  |  |  |  |
| Pearson Correlation | 0.986** | 0.991** | 0.518 | 0.939** |
| Sig. (2-tailed) | 0.000 | 0.000 | 0.189 | 0.001 |

**Correlation is significant at the 0.01 level (2-tailed), N = 8


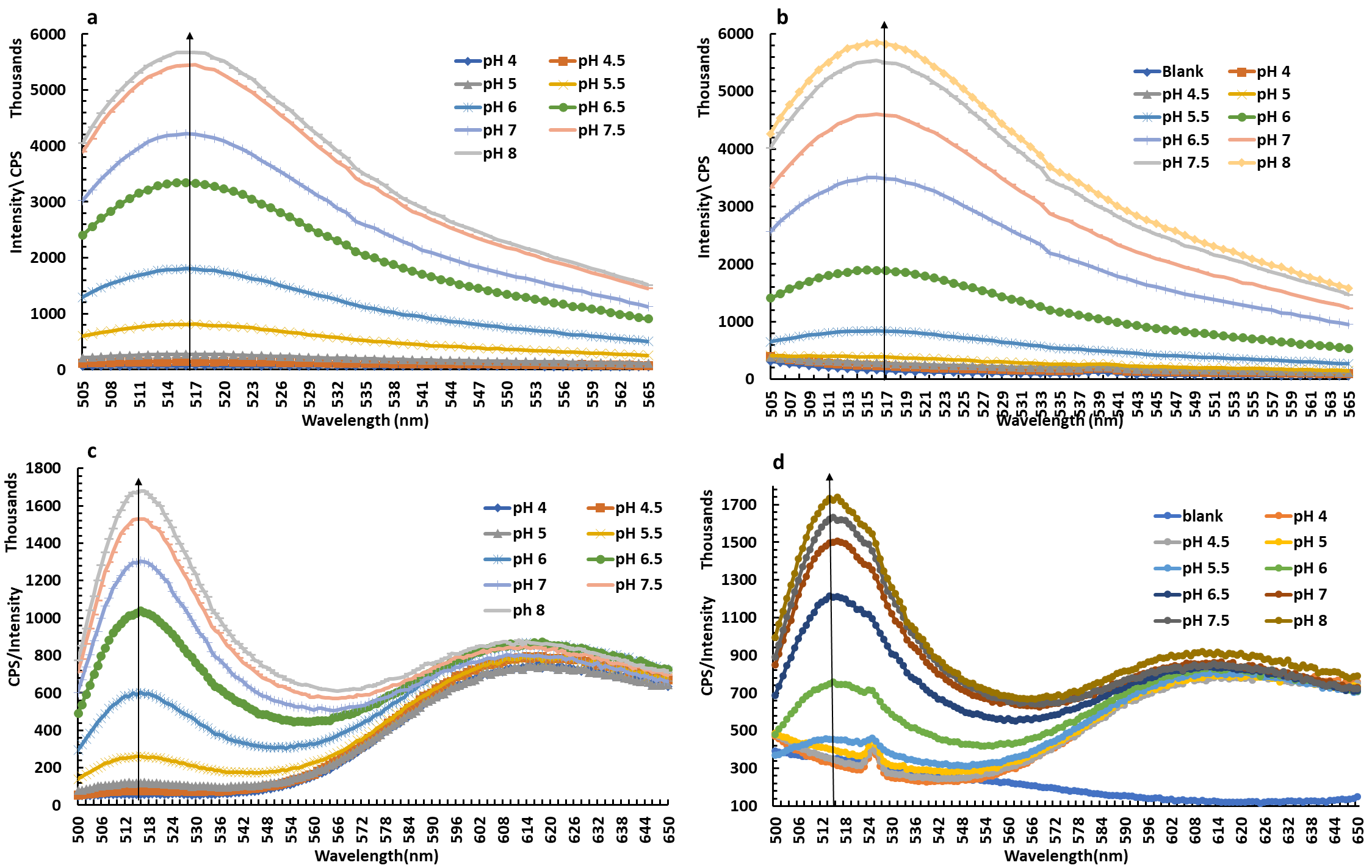


**Figure S1** Typical overlay of biosensor activity, AM-FD150 a) with standard pH buffer b) AM-FD150 with 1% milk sample; & AM-R-FD-150 c) with standard pH buffer d) with 1% milk sample

**Table S4** Accuracy studies of pH prediction using different pH biosensors when compared against a laboratory pH meter.

| **Type of Microspheres** | | **AM-FD500** | | **AM-R-FD500** | | | **AM-FD150** | | | **AM-R-FD150** | |
| --- | --- | --- | --- | --- | --- | --- | --- | --- | --- | --- | --- |
| **Actual pH** | **Predicted pH** | | **% Recovery** | **Predicted pH** | **% Recovery** | **Predicted pH** | | **% Recovery** | **Predicted pH** | | **% Recovery** |
| **7.5** | **6.9** | | **91.8** | **7.2** | **96.0** | **7.1** | | **95.3** | **7.2** | | **96.7** |
| **6.5** | **6.3** | | **97.5** | **7.0** | **107.3** | **6.8** | | **104.7** | **6.9** | | **107.1** |
| **5.5** | **5.4** | | **97.4** | **6.5** | **118.4** | **5.7** | | **104.2** | **6.0** | | **109.4** |
| **4.5** | **5.1** | | **113.3** | **6.6** | **146.1** | **4.9** | | **108.2** | **5.7** | | **127.7** |

**Table S5** Accuracy studies of pH prediction using different pH biosensors when compared against a laboratory pH meter during storage of raw milk.

| **Type of Microspheres** | | **AM-FD500** | |  | **AM-R-FD500** | | **AM-FD150** | | **AM-R-FD150** | |
| --- | --- | --- | --- | --- | --- | --- | --- | --- | --- | --- |
| **Time (hours)** | **Actual pH** | **Predicted pH** | **% Recovery** | **Actual pH** | **Predicted pH** | **% Recovery** | **Predicted pH** | **% Recovery** | **Predicted pH** | **% Recovery** |
| **00** | **6.8** | **5.0** | **72.8** | **7.3** | **7.3** | **100.2** | **6.8** | **92.6** | **7.9** | **107.9** |
| **06** | **5.5** | **5.7** | **103.5** | **6.7** | **6.7** | **100.8** | **6.1** | **90.8** | **7.2** | **108.4** |
| **12** | **4.9** | **5.0** | **102.1** | **5.9** | **6.1** | **101.9** | **5.0** | **84.7** | **6.1** | **103.1** |
| **18** | **4.8** | **4.9** | **101.7** | **5.2** | **6.2** | **119.4** | **4.5** | **87.1** | **5.6** | **108.2** |
| **24** | **4.7** | **4.9** | **102.0** | **4.8** | **6.1** | **125.7** | **4.6** | **95.1** | **5.4** | **110.6** |
